# Defining Boundaries for Ecosystem-Based Management: A Multispecies Case Study of Marine Connectivity across the Hawaiian Archipelago

**DOI:** 10.1155/2011/460173

**Published:** 2011

**Authors:** Robert J. Toonen, Kimberly R. Andrews, Iliana B. Baums, Christopher E. Bird, Gregory T. Concepcion, Toby S. Daly-Engel, Jeff A. Eble, Anuschka Faucci, Michelle R. Gaither, Matthew Iacchei, Jonathan B. Puritz, Jennifer K. Schultz, Derek J. Skillings, Molly A. Timmers, Brian W. Bowen

**Affiliations:** 1 Hawai’i Institute of Marine Biology, School of Ocean and Earth Science and Technology, University of Hawai’i at Mānoa, P.O. Box 1346 Kāne’ohe, HI 96744, USA; 2 Department of Zoology, University of Hawai’i at Mānoa, Honolulu, HI 96822, USA; 3 Department of Biology, Pennsylvania State University, University Park, PA 16802, USA; 4 Department of Biology, University of Hawai’i at Mānoa, Honolulu, HI 96822, USA; 5 Joint Institute for Marine and Atmospheric Research, University of Hawai’i at Mānoa, Honolulu, HI 96822, USA

## Abstract

Determining the geographic scale at which to apply ecosystem-based management (EBM) has proven to be an obstacle for many marine conservation programs. Generalizations based on geographic proximity, taxonomy, or life history characteristics provide little predictive power in determining overall patterns of connectivity, and therefore offer little in terms of delineating boundaries for marine spatial management areas. Here, we provide a case study of 27 taxonomically and ecologically diverse species (including reef fishes, marine mammals, gastropods, echinoderms, cnidarians, crustaceans, and an elasmobranch) that reveal four concordant barriers to dispersal within the Hawaiian Archipelago which are not detected in single-species exemplar studies. We contend that this multispecies approach to determine concordant patterns of connectivity is an objective and logical way in which to define the minimum number of management units and that EBM in the Hawaiian Archipelago requires at least five spatially managed regions.

## 1. Introduction

Global catches of commercially fished species have declined by up to 90% under classic single-species fisheries models [1–3]. The high-profile failures of fisheries managed for maximum sustainable yield has led to widespread interest in a shift toward ecosystem-based management (EBM) of marine resources (reviewed by [4]). EBM can be broadly defined as an integrated approach that considers the entire ecosystem, including linkages and the cumulative impacts of all human activities within and as part of the system. As such, EBM is explicitly place-based and adaptive in nature, and therefore particularly attractive for management. In recognition of the need for explicit boundaries in ecosystem-based management, Spalding et al. [5] divided the oceans into 232 ecoregions. However, marine ecosystems are highly complex, with many linkages and feedbacks that occur across multiple scales of space and time in ways that have proven difficult to predict [4]. Existing approaches to EBM in marine systems include spatial control of human activities through the use of marine protected areas (MPAs) and/or ocean zoning, changes in governance, monitoring and evaluation via ecosystem indicators derived from multiple disciplines (e.g., oceanography, ecology, economics, political science, and sociology), risk assessment, and precautionary adaptive management [6]. Successful spatial management requires a complex system of zones, each of which seeks to match resource exploitation with biological productivity, local population levels, and socioeconomic payoffs [7]. Delineation of the appropriate spatial scales for management zones within a specific management network requires a detailed understanding of dispersal pathways and population connectivity (reviewed by [8–10]). Despite the central role of dispersal and connectivity in sustaining marine populations, our understanding of these processes is still largely underdeveloped, and “a strong commitment to understanding patterns of connectivity in marine populations will clearly be necessary to guide the practical design of networks of marine reserves” [10, p.113]. In effect, managers cannot practice EBM if they do not know the boundaries of the corresponding ecosystems.

Understanding connectivity in the sea is complicated by the fact that most marine organisms have a biphasic life cycle with benthic or sedentary adults and dispersing eggs and/or larvae, which may be pelagic for as little as a few minutes to more than a year. Following the pelagic phase, larvae settle onto a patch of suitable habitat, where they may remain throughout their lives, and in cases of sessile organisms such as corals, the act of settlement includes permanent attachment to a single site. Thus, long-distance dispersal is accomplished almost exclusively during the pelagic larval phase, which can potentially span large expanses of open ocean [11–15]. On the other hand, species which lack a pelagic larval phase, such as marine mammals and elasmobranchs, have the potential to range widely throughout the oceans and face few obvious barriers to dispersal. Despite the potential for long-distance movement in most marine species, the geographic limits of such dispersal remain uncertain, because it is virtually impossible to track microscopic juveniles during the pelagic phase (reviewed by [16]), making indirect methods of quantifying larval dispersal particularly attractive (reviewed by [8, 17– 19]). Intuitive expectations that larval dispersal is a function of pelagic larval duration (PLD) are not supported by recent meta-analyses ([20–25]). Despite considerable research, the scale of larval dispersal and the boundaries for EBM remain nebulous due to the complex interaction of larval biology, oceanographic regimes, habitat quality and distribution, and the variability of each through time [26].

**Figure 1:**
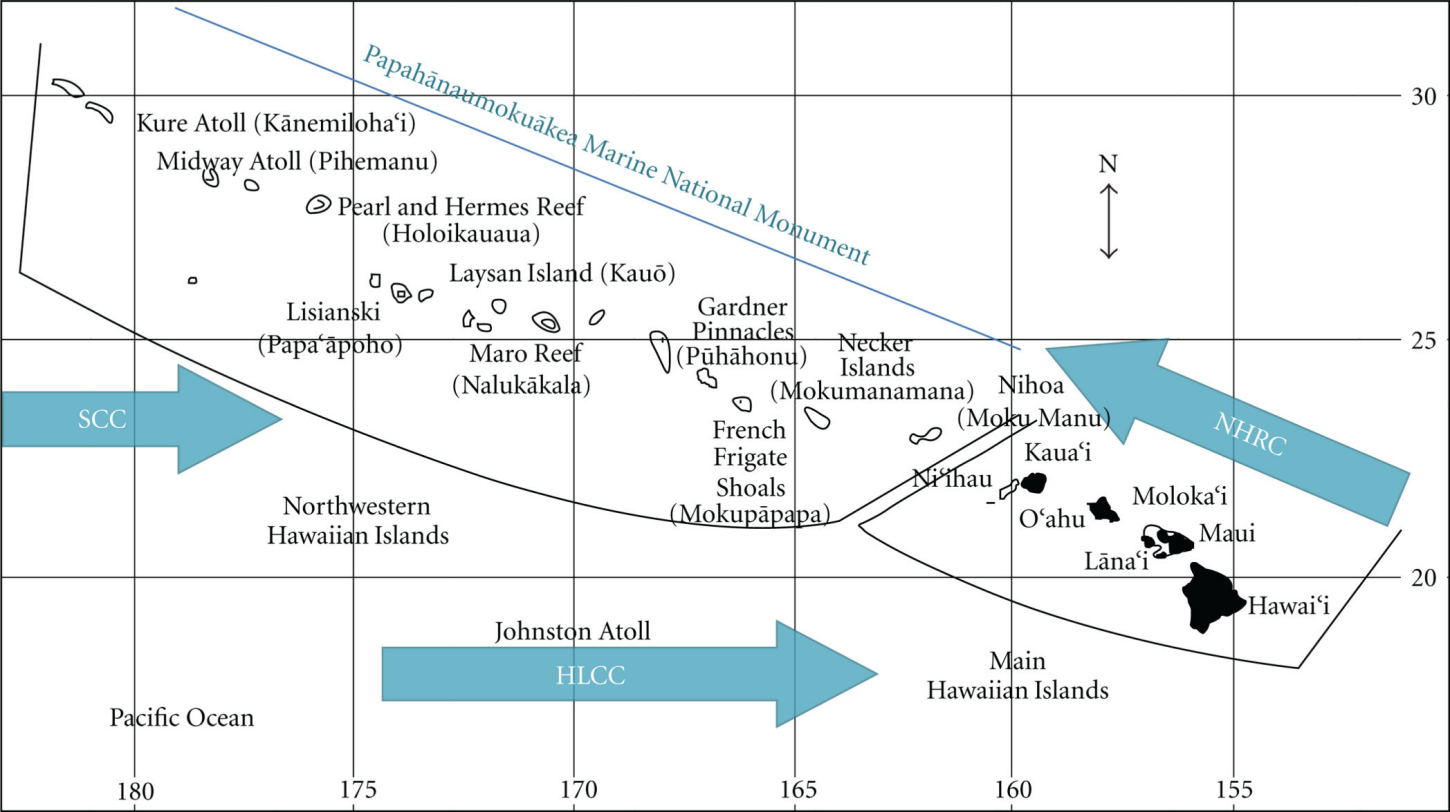
Map of the Hawaiian Archipelago with major currents denoted: the North Hawaiian Ridge Current (NHRC), the Hawaiian Lee Countercurrent (HLCC), and the Subtropical Countercurrent (SCC). The lines around the two regions of the archipelago highlight the islands, atolls, and banks protected within the Papahānaumokuākea Marine National Monument in the Northwestern Hawaiian Islands (NWHI) and the inhabited high islands of Main Hawaiian Islands (MHI) with each of the 15 primary target areas for collection labeled. For purposes of this analysis, the islands of Lāna’i, Maui & Moloka’i are treated as a single site within the Maui Nui complex of the MHI. Listed from northwest to southeast, these are: Kure Atoll (Kānemiloha’i), Midway Atoll (Pihemanu), Pearl and Hermes Reef (Holoikauaua), Lisianski (Papa’āpoho), Laysan Island (Kauō), Maro Reef (Nalukākala), Gardner Pinnacles (Pūhāhonu), French Frigate Shoals (Mokupāpapa), Necker Island (Mokumanamana), Nihoa (Moku Manu), Ni’ihau, Kaua’i, O’ahu, Maui Nui, and Hawai’i.

Delineating management units is further complicated by the fact that single-species studies of genetic connectivity are often contradictory. Analyses of connectivity frequently focus on single-species exemplars which are then extrapolated to the level of the community, but the utility of exemplars in such cases is limited; even among closely related species with similar ecology, life histories, and geographic ranges, the corresponding patterns of connectivity can be very different [27, 28]. In other cases, animals with highly divergent biology can have surprisingly similar patterns of connectivity [26]. Such variability among species appears to be the rule rather than the exception, and has led to a call for multispecies comparisons of connectivity across trophic levels to broadly define the boundaries for management, and to determine shared avenues of exchange among ecosystems. Due to logistical difficulties in completing such comparisons in marine habitat, few such studies exist (e.g., [29–31]). The linear nature of the Hawaiian Archipelago (Figure 1) provides an excellent forum for resolving shared barriers to gene flow across species and trophic levels, with the goal of developing a geographic framework for EBM.

The Hawaiian archipelago stretches more than 2600 km in length and consists of two regions: the Main Hawaiian Islands (MHI) which are high volcanic islands with a heavy human presence and the Northwestern Hawaiian Islands (NWHI) which are a string of tiny islands, atolls, shoals, and banks that are essentially uninhabited. Due to their isolation, the roughly 4,500 square miles of coral reefs in the NWHI are among the healthiest and most extensive remaining in the world [32] with abundant large apex predators, a high proportion of endemic species [33, 34], and few human impacts compared to the MHI [18, 35]. In contrast, coral reefs in the MHI are under considerable anthropogenic pressure from the 1.29 million residents (with over 900,000 of those living on the island of O’ahu) and the more than 7 million tourists that visit the state annually. Coral reefs in many of the urban areas and popular tourist destinations have sustained significant impacts, and many show ongoing declines [35– 37]. The primary impacts to coral reefs in the MHI are local and anthropogenic, including coastal development, land-based sources of pollution, overfishing, recreational overuse, and alien species. In contrast, the primary stressors in the NWHI are global in nature, including climate change, ocean acidification, and marine debris [18, 35–37].

On June 15, 2006, the President of the United States signed a proclamation creating the Papahānaumokuākea Marine National Monument (PMNM), encompassing the entire NWHI, at the time the world’s largest marine protected area (MPA). The monument designation affords the NWHI the greatest possible marine environmental protection under United States law. The PMNM spans nearly 140,000 square miles and is home to more than 7,000 currently described species including fishes, invertebrates, algae, marine mammals, and birds although many biologists believe that this is a gross underestimate of the true biodiversity in the region [38]. While the full extent of PMNM biodiversity is unknown, about 25% of the known species are found nowhere else on Earth [39–42]. In 2010, the PMNM was inscribed to the UNESCO World Heritage List, the first U.S. site to be designated in over 15 years. The remote PMNM and surrounding waters became the first primarily marine site to be named in the United States, and the first primarily marine location in the world to be designated as a mixed site for both its outstanding natural and cultural value.

Our research group has embarked on a genetic survey of approximately 60 species of reef-associated fishes, gastropods, crustaceans, echinoderms, cnidarians, and marine mammals, designed to address the issue of population connectivity across the Northwestern and Main Hawaiian Islands and linkages of the Hawaiian Archipelago to other locations throughout the Central Pacific. This effort seeks to inform ecosystem-based management of the PMNM and to evaluate the potential for spillover from the protected area of the NWHI to the heavily populated and exploited MHI. Here we take a molecular genetic approach to infer patterns and magnitude of connectivity in a suite of taxonomically diverse reef-associated species and present preliminary results from 27 species, a subset of the 60 or so target species being collected to understand connectivity across the Hawaiian Archipelago and aid in defining the spatial scale over which EBM should be considered. Although EBM is explicitly place-based, and superficially the definition of an ecosystem seems straightforward, the resolution of geographic boundaries is confounded by obscure biological and oceanographic processes in most marine locations that complicates direct application to management (reviewed by [43]). In managing reefs in the Hawaiian Archipelago, what exactly constitutes a coral reef ecosystem? Is it a reef complex, an island or atoll, an arbitrary geographic distance, a series of adjacent islands and atolls, or the entire Archipelago that is the appropriate geographic scale for management? This work seeks to resolve and quantify the direction and magnitude of exchange among reef habitats across a broad taxonomic spectrum, and to use this information to define objective boundaries, as a necessary prerequisite for the implementation of EBM.

## 2. Methods

### 2.1. Sample Collection, DNA Extraction, and Amplification

Tissue samples for DNA analyses were collected from approximately 60 species at as many of the 16 primary islands and atolls as possible in the Hawaiian Archipelago, including the remote and tightly regulated NWHI (Figure 1). It is important to note that sampling remote areas of the Pacific is difficult and expensive and requires extensive permitting and voyage planning compared to collections on the mainland; permitted collections are limited to a maximum of 50 individuals per species at each site, and there are only 1 or 2 days per location, during which the researchers are at the mercy of the weather as to whether or not they can even launch dive boats. Thus, we do not have complete coverage for all species, but in addition to the two species available in the published literature (e.g., [44]), we have currently analyzed 25 additional species (total 27, Tables 1 and 2) from many of the islands and atolls across the Hawaiian Archipelago (Figure 1). Details for the sampling protocols, tissue preservation, DNA extraction, and amplification can be found in Iacchei and Toonen [45] and Skillings and Toonen [46]. Briefly, tissue biopsy samples were taken in the field and stored in either 20% dimethyl sulfoxide salt-saturated buffer [47] or >70% ethanol. DNA was extracted using either a commercially available extraction kit (e.g., Qiagen DNeasy), the chloroform extraction protocol described in Concepcion et al. [48] or a modified salting-out protocol [49]. Following extraction, DNA was stored at −20°C. Most studies were conducted with direct sequencing of a mtDNA fragment using the polymerase chain reaction (PCR) as outlined in references from Table 1. In general, a segment of approximately 600–800 base pairs of the mtDNA cytochrome *b* (Cytb) was amplified from most of the fishes, and cytochrome oxidase subunit I (COI) was amplified from the majority of invertebrate species, but some used other mitochondrial or nuclear sequence regions or microsatellite markers (see Table 1 for details). PCR recipes and cycling conditions for individual species are provided in the publications cited in Table 1 and upon request from the authors. PCR products to be sequenced were treated with 1.5 units of Exonuclease I and 1.0 units of Fast Alkaline Phosphatase (ExoFAP, Fermentas) per 15 µL PCR products at 37°C for 60 minutes, followed by deactivation at 80°C for 10 minutes. DNA sequencing was performed with fluorescently–labeled dideoxy terminators on an ABI 3130XL Genetic Analyzer (Applied Biosystems) at the Hawai’i Institute of Marine Biology EPSCoR Sequencing Facility. All specimens were initially sequenced in one direction and unique genotypes were confirmed by sequencing in the opposite direction. For analysis of microsatellite loci, amplification products were visualized on an ABI 3130XL Genetic Analyzer using GS500LZ size standards, and analyzed using Genemapper 4.0 (Applied Biosystems).

### 2.2. Genetic Analyses

**Figure 2:**
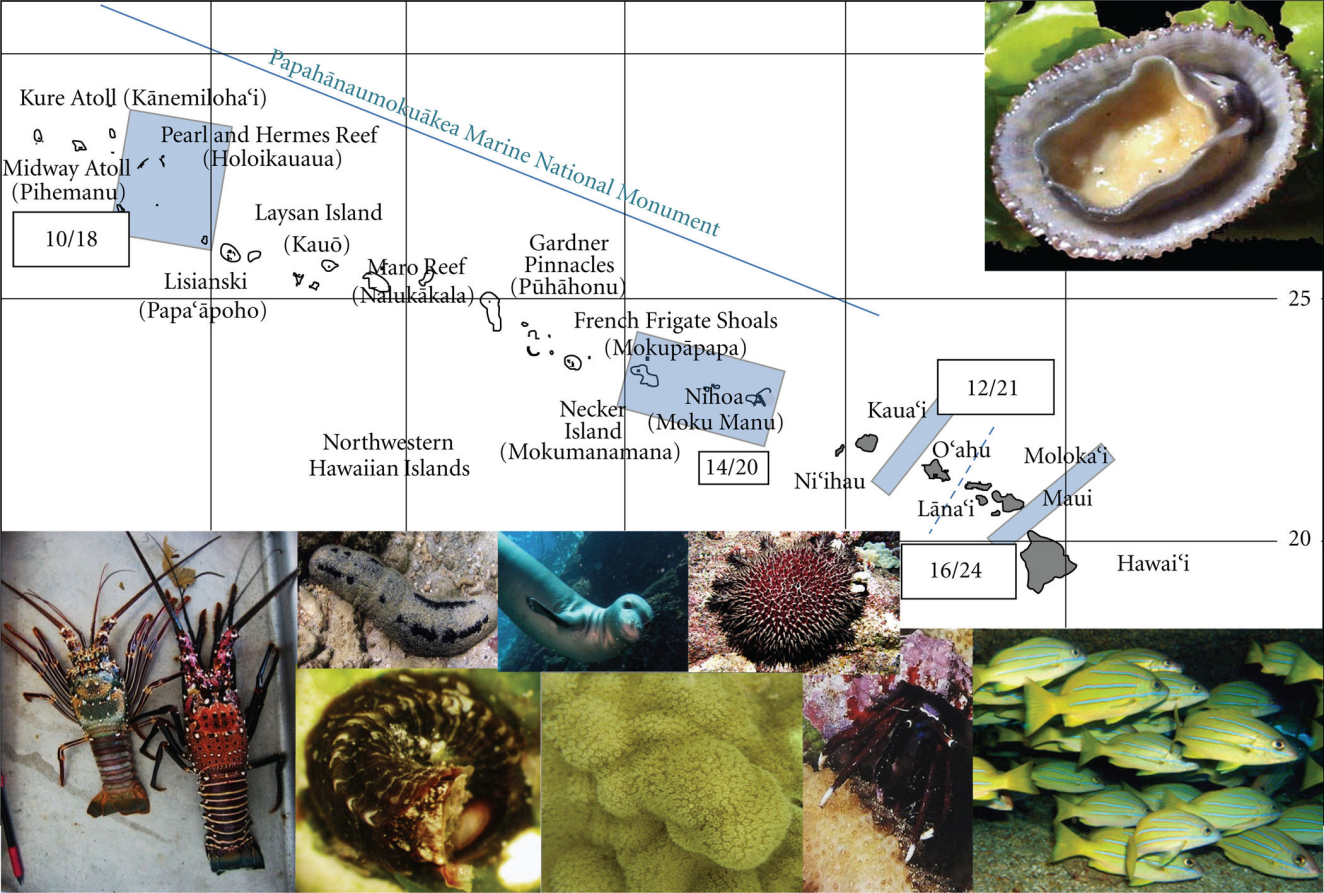
Map of the Hawaiian Archipelago with significant consensus genetic breaks among the 27 taxa listed in Table 1 overlaid as blue bars between islands. In each bar, the number of species that show evidence for restricted gene flow across the barrier is listed in the numerator, and the total number of species for which we have data across that geographic area is listed in the denominator. The total number of sites included for each species is variable because not all species have been collected or analyzed at each site. The dotted line between Maui Nui and O’ahu highlights the location of the barrier that is shared by 8 of the surveyed species but is not significantly different than random expectations. The images include some of the species included in these analyses (left to right): *Panulirus penicillatus, Panulirus marginatus, Holothuria atra, Dendropoma rhyssoconcha, Monachus schauinslandi, Porites lobata, Acanthaster planci, Calcinus hazletti, Lutjanus kasmira, and Cellana sandwicensis* (photo credits to Derek Smith, Joe O’Malley, and the authors).

For each species, details of the analyses are provided in the studies cited in Table 1, or upon request from the authors. In brief, overall genetic variation was partitioned among sites as pairwise Φ_ST_ using the best fit model of sequence evolution, as determined by Modeltest 3.7 [62], that could be implemented by Arlequin 3.11 [63]. For most of the studies, *F*_ST_ was standardized for within population levels of heterozygosity [64, 65], and calculations of *D*_est_ [66] were done manually using formula macros in Microsoft Excel ([67] in review). The number and location of shared genetic breaks among species across the Archipelago are unchanged whether corrected or uncorrected *F*- statistics or *D*_est_ was used because the relative differences between these values are all highly correlated with our data set (data not shown). Because any set level of divergence selected is ultimately arbitrary, we use a significant pairwise *F*_ST_ among populations sampled on either side of the channel of interest as our metric of divergence. Significance of pairwise values was determined by permutation testing in Arlequin, with False Discovery Rate (FDR) correction for multiple tests [68] unless otherwise specified in the original publication (Table 1). Significant pairwise differences among adjacent islands, were overlaid visually on a map of the Hawaiian Archipelago (Figure 1) species-by-species. The number of significant pairwise differences among locations was summed across all 27 species and those that exceed random expectations (see below) are depicted in Figure 2.

### 2.3. Statistical Testing of Shared Genetic Barriers

Because not all species are collected in all locations, we looked only at the channels between adjacent islands where samples of that species were available on both sides so that a test for pairwise population differentiation was possible at that site. We initially excluded any sites for which there were fewer than 20 individuals from each location on adjacent sides of the channel being tested, but found that the presence and location of barriers was unaffected in these analyses with any sample size greater than 5 individuals per site (data not shown). Thus, in the interest of including as much data as possible in this comparison, we include all sites for which the sample size was 5 or more (Table 2). We observed a total of 73 significant pairwise differences among the 178 possible pairwise tests for these species (Tables 1 and 2). The distribution of these pairwise differences was tested using a χ^2^ test with 13 degrees of freedom (14 between island channels); we calculate the expected number of the 73 pairwise differences that would occur, weighted by sample size between each island, at random within each channel. The validity of a shared genetic break at any given location was also tested using a χ^2^ to determine if the number of observed significant pairwise differences across the species sampled at that location differed significantly from the null expectation that all detected breaks were distributed equally among the 14 interisland channels.

## 3. Results

Although each species differs in the particular pattern of population structure and the inferred magnitude of larval exchange among sites, some consistent genetic breaks are apparent among these divergent species (Figure 2). In particular, the data indicate four strong barriers to gene flow in the channels between: (1) the Big Island of Hawai’i and Maui, (2) the islands of O’ahu and Kaua’i, (3) the MHI and NWHI, and (4) the far NW end and the rest of the NWHI chain around Pearl and Hermes Reef (Figure 2). The presence or absence and the strength of a given barrier vary among species (see references in Table 1). Likewise, there are some significant barriers that appear for only one or a few species, but do not appear in the majority of study organisms (e.g., Laysan Island for the sea cucumber, *Holothuria atra*, see Skillings et al. [56], or Gardner Pinnacles for the endemic grouper *Epinephelus (=Hyporthodus) quernus*, see Rivera et al. [51]).

Despite the vast differences in natural history among taxa, more than 50% of the species surveyed to date share the same four concordant barriers to gene flow across the Hawaiian Archipelago (Figure 2). Notably 8 of 19 species also show a break between O’ahu and Maui Nui, but this partition is not significantly different from random expectations (χ^2^ = 0.17, df = 1, *p* > 0.05). Essentially, roughly 50% of the sampled species must share a genetic discontinuity in order to deviate from the random expectation of 5.2 significant differences in each channel (χ^2^ = 4.4, df = 1, *p* < 0.05). Other than the four significant breaks depicted in Figure 2, and the nonsignificant split between O’ahu and Maui Nui, no other inter-island channel constitutes a barrier for more than 4 of the sampled species. Thus, with the caveat that additional sampling may yet demonstrate a fifth significant barrier between O’ahu and Maui Nui, there are currently four significant shared barriers to gene flow that divide the Hawaiian Archipelago into a minimum of five distinct ecoregions with limited exchange. In stark contrast to those locations, other inter-island channels have significantly fewer barriers than expected by chance (e.g., the region between French Frigate Shoals and Pearl & Hermes Atoll in the NWHI, χ^2^ = 3.85, df = 1, *p* = 0.05). This overall pattern of high connectivity among some locations and shared genetic barriers in others across the archipelago is significantly nonrandom (χ^2^ = 56.18, df = 13, *p* < 0.0001).

Distance alone is a poor predictor of the locations of these barriers to dispersal. The distance between areas that are isolated can be quite small (such as the ‘Alenuihāhā Channel between Hawai’i and Maui, ~45 km) whereas much larger distances between atolls in the NWHI generally show no consistent barriers to dispersal (for example Gardner Pinnacles is ~180km northwest of French Frigate Shoals). Likewise, more of the significant barriers to dispersal are found in the geographically smaller (600 km) MHI with the significant absence of barriers occurring in the geographically larger (2000 km) NWHI. Because adjacent sites can be highly differentiated whereas more distant sites are not, relatively few species (7/27) show a significant signal of isolation-by-distance across the Hawaiian archipelago (see Table 2 for highlighted exceptions).

## 4. Discussion

These data are striking in that more than half of the species surveyed show significant concordant barriers to gene flow concentrated in the four highlighted regions of the Archipelago (Figure 2). Given the broad differences in taxonomy, life history, and ecology of the species surveyed, including limpets, sea cucumbers, vermetid tube snails, reef fishes, monk seals, and spinner dolphins (Table 1), there is no *a priori* reason to expect that patterns of connectivity would be shared among the majority of the species. However, the four shared barriers to dispersal highlighted here indicate that these species are responding to common factors that limit dispersal and delineate independent units in terms of connectivity over management-relevant time scales. We hypothesize that the dominant factors are likely abiotic as opposed to biotic, given the diversity of species with radically divergent life histories that share the pattern of isolation.

### 4.1. Discordance between Genetic and Oceanographic Predictions

The most obvious candidates for such physical barriers to gene flow are geographic distance and oceanic currents. For most species there are enigmatic restrictions to dispersal that appear to have little to do with geographic distance. Many of the studies listed in Table 1 provide cases of divergence among proximate sites in the face of lower divergence among more distant sites elsewhere in the archipelago. Regardless, the overall dataset indicates that much of the NWHI is well connected despite greater average distances among the sites whereas the MHI show greater structure on average despite geographic proximity. Although some species do show isolation-by-distance, there appears to be a substantial taxonomic effect because three of the seven cases are sister species of *Cellana* limpets, and two of the remaining four are scleractinian corals (Table 2). While we cannot rule out the role of distance in limiting dispersal within the Hawaiian Archipelago, the impact of distance on the probability of dispersal does not appear to be a simple linear effect for the majority of species surveyed to date. This discord is not particularly surprising given the complexity of oceanographic current patterns. Recent analyses of larval dispersal in the Southern California Bight showed that probability of exchange among sites was uncorrelated with geographic distance, but strongly correlated with a derived “oceanographic distance” including realistic annual water movement patterns across many years [26, 69].

In Hawai’i, however, the patterns of genetic differentiation do not generally match predictions for larval dispersal based on water movement information from either a two-dimensional Eulerian advection-diffusion model [70, 71] or a Lagrangian particle-tracking model [72, 73]. One of the primary predictions of both simulation models is that the average distance of larval dispersal is short, roughly on the order of 50–150 km, and that the Main Hawaiian Islands (MHI) ought to be consistently connected and well mixed whereas the NWHI ought to show a number of isolated populations [70, 71]. For a PLD of less than about 45 days, the larval dispersal simulations predict a majority of local recruitment of larvae to their natal island/atoll or the adjacent ones (see [51]). In stark contrast to the primary prediction of the available larval dispersal simulation models (a well-mixed MHI and comparatively patchy NWHI), the consensus finding across 27 taxa to date is the opposite: the MHI show far more population structure than any equivalent geographical scale within the NWHI, and the primary dispersal barrier predicted by Eulerian simulation models is located in the region of the NWHI in which there is a significant paucity of population structure among surveyed species (Figure 2). Possible reasons for oceanographic simulations failing to predict the structure observed in the empirical genetic data are many (reviewed by [22, 74, 75]), but given the number and diversity of taxa across which the pattern holds, the genetic inference of isolation between the four regions highlighted in Figure 2 is robust.

### 4.2. Multispecies Approaches to Measuring Connectivity

All connectivity studies face practical limitations in terms of the number of specimens, sample sites, and taxonomic scope of study, which is why the vast majority of studies to date have focused on one or a few exemplar species to draw generalizations. Exemplar species are an attractive compromise to guide conservation and management efforts given the imposing logistic and resources challenges of conducting connectivity studies on all species of management relevance. However, the utility of exemplar species depends on whether they represent the community as a whole. Unfortunately, in most cases where this assumption has been tested explicitly, the patterns of dispersal and genetic structure differ significantly and unpredictably even among closely related species with similar life histories (e.g., [28, 52, 54, 76–78]). Despite the perceived potential for long-distance dispersal and broad mixing in the ocean, many taxa show unique archipelagic diversity (e.g., [79]) and even finer scale population structure than expected (e.g., [31, 80]). Regardless of whether we compare within taxonomic groups or between them, some of the species we have surveyed (e.g., *Myripristis berndti*, *Centropyge loricula*, *Lutjanus kasmira*, *Acanthaster planci*, and *Calcinus* spp.) appear to live up to their expected potential for dispersal and show no significant population structure across the Central Pacific (see [54, 57, 81–84]). In contrast, other species that appear capable of extensive dispersal (*Epinephelus quernus*, *Ctenochaetus strigosus*, *Stenella longirostris*, and *Zebrasoma flavescens*) show significant population differentiation within the Hawaiian Archipelago [50, 52, 53, 61, 85] and island-by-island or in some cases even site-by-site differences in population structure (e.g., [28, 44] Faucci et al. unpubl. data). Despite the potential for wide dispersal, Christie et al. [85] use individual parentage analyses to document self-recruitment in the Yellow Tang (*Zebrasoma flavescens*) and illustrate that at least some larvae recruit to the same region of the Kona coastline from which they were originally spawned.

Such variability among species greatly complicates efforts to generalize management implications from single-species studies and severely restricts the utility of exemplar species for decision making in conservation and management. While there is a consistent push to move beyond single-species management plans and implement EBM at a national and international level (e.g., [86, 87]), the exact geographic scale at which EBM should be applied is seldom obvious, and the accumulating data indicate that studies of marine connectivity cannot be generalized easily for this purpose. It is clearly impractical to study every species individually, and even if we could, how would the connectivity matrix from all those species be combined into a single coherent data set to guide EBM? For example, the multispecies conservation plan for U.S. federal lands states: “conservation objectives will not be achieved with a single reserve or a single population. Rather, local populations widely scattered across the landscape, but connected by movement, will be necessary. Few of these populations will be large enough to avoid problems faced by small populations, such as extirpation due to stochastic factors and inbreeding depression. Connectivity maintenance is therefore one of the most critical aspects of multispecies conservation. Connectivity, however, is notoriously difficult to directly measure” [88, p.64]. A variety of landscape genetic approaches to identifying cryptic barriers to connectivity have been proposed (e.g., [69, 89, 90]), but with few exceptions (e.g., [26]) such work has also been conducted on single-species. The push to implement EBM highlights an explicit need for multi-species comparisons of connectivity across all trophic levels to define the boundaries for management and resolve shared avenues of exchange among ecosystems.

Due to resource constraints as well as the logistical difficulties in completing such multispecies comparisons, only a few such studies exist. The few explicit multispecies connectivity studies that have been conducted to date (e.g., [29–31]) all face the limitation that there is no generally accepted method by which to analyze the aggregate connectivity data. Thus, like the study presented here, the primary method of analyzing shared genetic breaks is by counting the number or proportion of species that share a genetic discontinuity among locations. For example, a survey of 50 coastal marine species along the west coast of North America concluded that a greater proportion of species show significant genetic differentiation between the central (40% of species between Monterey, CA and Cape Blanco, OR) and northern sites (33% of species between Cape Blanco and Sitka, AK) than between the southern sites (15% of species between Monterey and Santa Barbara, CA; [30]). Likewise, a survey of 9 species of fish and 10 species of invertebrates in Indonesia defines partitions where more than two or three species share a phylogenetic break [31]. We have employed a similar approach with counting up shared genetic discontinuities in the data set, but elected to test whether these shared breaks deviate significantly from random. In our study, a surprisingly high number of species need to share a break to deviate significantly from random: even where 8 of the 19 species show differentiation between O’ahu and Maui Nui, that result was non-significant. The overall pattern of genetic divergence among sites within the Hawaiian Archipelago is highly non-random, with the central region of the NWHI having significantly fewer genetic breaks, and four individual channels emerge as having significantly more species sharing a break than expected at random (Figure 2).

There are substantial caveats to comparing *F*_ST_ and Φ_ST_ values directly among studies and marker classes (reviewed by [91–93]). Further, several recent publications have pointed out that the maximum attainable *F*_ST_ is inversely proportional to the mean within-population heterozygosity [64, 65], and therefore does not accurately measure population differentiation [66]. Thus, for highly polymorphic genetic markers, such as microsatellite loci, the maximum attainable *F*_ST_ is reduced far below one [64]. Contrary to the intuition that more polymorphic loci will reveal finer population structure, *F*_ST_ values are actually constrained to be lower as allelic diversity gets higher [67]. This limitation has led some to advocate the use of “true genetic differentiation” (*D*_est_) as the primary or only means of comparison (e.g., [66]). While an attractive alternative in theory, there is as yet no means of significance testing for *D*_est_, and researchers have to pick an arbitrary value at which to determine a genetic break before comparisons can be made; however, in the absence of statistics any cutoff value selected can be arbitrary and problematic [94]. For example, Kelly and Palumbi [30] chose Φ_ST_ = 0.10 as the delineation between strong (Φ_ST_ = 0.11–0.60) and moderate (Φ_ST_ = 0.02–0.10) population structure. While there is nothing wrong with this delineation, one could have also chosen Φ_ST_ = 0.05 or Φ_ST_ = 0.15 with equal justification, and there is no consistent and defensible level of population structure that determines the cut-off at which management decisions ought to be made [60]. Most published estimates of population structure remain uncorrected for marker variation and heterozygosity; thus, a value of 0.10 in one species may be on a completely different scale than in the next species if they have different levels of mean within population heterozygosity [64, 66, 67]. For this reason we use statistical significance as our cutoff and draw no inferences regarding the magnitude of the barriers beyond the number of species that share them. A method by which the boundaries of an ecosystem can be defined with multi-species data sets, and linkages between ecosystems can be quantified, is a logical prerequisite for successful implementation of EBM in the sea.

### 4.3. Connectivity in the Hawaiian Archipelago: Not 1 But at Least 5 Distinct Regions

The primary finding of this work is that the Hawaiian Archipelago is not a single, well-mixed community, but rather there are at least four significant multi-species barriers to dispersal along the length of the island chain. Additional sampling or more sophisticated statistical analyses may reveal additional barriers, but we report four strong concordant breaks here. As outlined above, some species cross these barriers, others do not, and the patterns of connectivity can, and do, vary dramatically among individual species (see refs. in Table 1). Regardless, a strong and consistent pattern emerges from the multispecies comparison in which the majority of 27 taxonomically diverse species share four significant concordant genetic breaks across the archipelago. It is noteworthy that the variability among individual species studies of connectivity published to date certainly does not lend itself to an expectation of such strong concordant patterns. Despite the suite of taxonomic, ecological, and biological differences that might lead us to expect highly divergent patterns among these diverse taxa, some unknown barriers appear to consistently limit dispersal in a majority of the 27 species surveyed to date. These results illustrate that while a single species is rarely representative of the average connectivity, concordant patterns can emerge when many species are examined simultaneously. Insofar as this is a general result, it would mandate that a broad suite of reef species across multiple taxonomic groups and ecological niches ought to be surveyed to resolve general trends and to provide connectivity information pertinent to management of any large marine management area such as the PMNM.

The two primary caveats to this finding are that: (1) the basis for these shared genetic restrictions is poorly understood and discovering the location of these barriers is only the first step, and (2) it is an overly simplistic statistical model to show significant deviations from random pairwise differences across species as a measure of the strength of dispersal barriers. Nonetheless, such summing is the primary means of comparison available at this time, and this is the only multispecies study that employs even this simplistic statistical approach. In terms of the first caveat, it will be valuable to determine the ecological and oceanographic factors driving regional, island, or site specific genetic structure; this will likely be important for ecosystem-based management of both the Main and Northwestern Hawaiian Islands, and may provide general characteristics to predict ecosystem-level partitions among coral reefs elsewhere. Discovering the existence and location of these barriers leads to questions about the underlying cause for so many species sharing these concordant patterns, and what maintains those barriers to dispersal among taxa as diverse as limpets and dolphins. In terms of the second caveat, as outlined above, we need to develop a quantitative method for multispecies studies of connectivity among many locations. Ultimately, it would be ideal to bring the multispecies data sets together in a single analysis to determine both the relative strength and statistical confidence in each of the detected barriers, but no such method exists currently.

### 4.4. Conclusions and Management Implications

This multi-species approach to understanding population connectivity across the Hawaiian Archipelago reveals four previously unrecognized barriers to dispersal that delineate five relatively isolated regions of the Hawaiian Archipelago. In contrast to predictions based on either geographic distance between islands (isolation by distance) or on larval dispersal model predictions using pelagic larval duration, there are more barriers to dispersal within the Main Hawaiian Islands (MHI, ~600 km) than the Northwestern Hawaiian Islands (NWHI, ~2000 km). The underlying mechanism of this isolation remains unknown, but the concordance across 52% to 70% (depending on the barrier) of the 27 taxonomically and ecologically divergent species sampled here demonstrates that the pattern is robust and likely to derive from physical rather than biologically intrinsic factors.

These data provide information pertinent to current management issues facing the broader Pacific and efforts to implement ecosystem-based management (EBM) in Hawai’i. In particular, these data directly address the controversy about whether the NWHI is a series of isolated (and therefore relatively fragile) island ecosystems, and whether the Papaphānaumokuākea Marine National Monument provides spillover benefits to the highly exploited waters of the MHI [35]. We find that the NWHI are far more connected on average (and therefore comparatively robust) than the MHI, but that connectivity between the MHI and NWHI is limited. The results highlight that the Main Hawaiian Islands are isolated in terms of resource management and will not receive substantial subsidy from the Papahānaumokuākea Marine National Monument; the MHI must stand alone in management of marine resources. Furthermore, even the comparatively small MHI are not a single panmictic unit, and future management plans should incorporate knowledge of the substantial isolation among multiple regions within the MHI. For example, Bird et al. [28] argue that each island should be considered a separate management unit for the culturally important Hawaiian limpets (‘opihi, genus *Cellana*). Likewise, the impact of invasive species is felt globally and with 343 alien marine species documented in Hawai’i thus far [95], there is considerable concern regarding the vulnerability of Hawaiian reefs to invasion and the likely spread of aliens that are already introduced. Our findings predict barriers through which invasive species should have difficulty advancing, and indeed recent studies of several species of invasive fishes and invertebrates appear to corroborate those predictions (e.g., [96, 97].

This study is one of the few multispecies surveys of marine connectivity to date and confirms that this approach can illuminate general patterns pertinent to management that do not emerge from single-species exemplar studies. The manner in which policy makers delineate the boundaries for ecosystem-based management remains a subject of considerable debate, but we argue this multispecies approach offers a possible solution. Here, we resolve concordant patterns of connectivity in an objective and quantitative manner to define a minimum of five marine spatial management units in the Hawaiian Archipelago.

## Figures and Tables

**Table 1:** Species of marine organisms, total sample size, total number of sites, genetic marker(s) used, and study citation for each of the organisms surveyed for population genetic structure across the Hawaiian Archipelago to date. Not all samples were included in subsequent analysis, therefore, the actual sample sizes by site for each species in this analysis are provided in Table 2 . Abbreviations for genetic markers used are: SSR = microsatellites; NIS = nuclear intron sequence data; Cytb = cytochrome *b*; COI = cytochrome oxidase subunit I; COII = cytochrome oxidase subunit II; CR = control region.

Species name	Sample size	Number of sites	Marker	Reference
Fishes:				
(1) *Epinephelus* (=*Hyporthodus*) *quernus*	301	10	SSR, CR	Rivera et al. (see [50, 51])
(2) *Stegastes fasciolatus*	219	7	CR	Ramon et al. [44].
(3) *Dascylus albisella*	102	7	CR	Ramon et al. [44].
(4) *Ctenochaetus strigosus*	499	15	Cyt*b*	Eble et al. [52].
(5) *Zebrasoma flavescens*	528	15	Cyt*b*	Eble et al. [52, 53].
(6) *Acanthurus nigrofuscus*	305	11	Cyt*b*	Eble et al. [52].
(7) *Lutjanus kasmira*	385	9	Cyt*b*, NIS	Gaither et al. [54].
(8) *Squalus mitsukurii*	112	6	CR	Daly-Engel et al. [55].
Gastropods:				
(9) *Cellana exarata*	150	7	COI	Bird et al. [28].
(10) *Cellana sandwicensis*	109	6	COI	Bird et al. [28].
(11) *Cellana talcosa*	105	5	COI	Bird et al. [28].
(12) *Dendropoma gregaria*	176	15	COI	Faucci et al. (unpubl. data)
(13) *Dendropoma platypus*	143	15	COI	Faucci et al. (unpubl. data)
(14) *Dendropoma rhyssoconcha*	94	11	COI	Faucci et al. (unpubl. data)
(15) *Serpulorbis variabilis*	73	13	COI	Faucci et al. (unpubl. data)
Crustaceans:				
(16) *Calcinus haigae*	146	5	COI	Baums et al. (unpubl. data)
(17) *Calcinus hazletti*	179	12	COI	Baums et al. (unpubl. data)
(18) *Calcinus seurati*	161	4	COI	Baums et al. (unpubl. data)
(19) *Panulirus marginatus*	449	14	COII	Iacchei et al. (unpubl. data)
(20) *Panulirus penicillatus*	227	9	COI	Iacchei et al. (unpubl. data)
Echinoderms:				
(21) *Holothuria atra*	399	15	COI	Skillings et al. [56]
(22) *Holothuria whitmaei*	427	10	COI	Skillings et al. (unpubl. data)
(23) *Acanthaster planci*	338	11	CR	Timmers et al. [57]
Scleractinian:				
(24) *Montipora capitata*	551	13	SSR	Concepcion et al. (unpubl. data)
(25) *Porites lobata*	443	11	SSR	Polato et al. [58]
Marine Mammals:				
(26) *Monachus schauinslandi*	2409	8	SSR	Schultz et al. [59, 60]
(27) *Stenella longirostris*	386	8	SSR, CR	Andrews et al. [61].

**Table 2:** Sample size per location (refer to Figure 1 for Hawaiian names of sites) for each of the 27 species included in the combined multispecies analysis of population genetic structure across the Hawaiian Archipelago to date. Locations with fewer than 5 individuals were excluded from all analyses and are not included here (see text).

Species Name	Hawai’i (=Big Island)	Maui Nui	O’ahu	Kaua’i	Ni’ihau	Nihoa	Necker Island	French Frigate Shoals	Gardner Pinnacles	Maro Reef	Laysan Island	Lisianski	Pearl & Hermes Reef	Midway Atoll’	Kure Atoll
*Hyporthodus* (*=Epinephelus*) *quernus*	36	30	9	30		44	30		30	46		29	27		
*Stegastes fasciohtus*	27		42	49				24					15	20	42
*Dascylus albisella*	10		8					25					28	7	14
*Ctenochaetus strigosus*	102	100	40	28		29		37	29	31			33	32	38
*Zebrasoma flavescens	146	122	35	42		20		40		26			29	33	35
*Acanthurus nigrofuscus*	33	65	39	26		39		33	28				28	6	9
*Lutjanus kasmira*	101	39	50	36			49	40		21				40	9
*Cellana exarata	41	18		21		11	23	36							
*Cellana sandwicensis	42	21		20			18	8							
*Cellana talcosa	43	24		38											
*Dendropoma gregaria*	53	25	39	20			5	5	5	5		5	5		5
*Dendropoma platypus*	16	20	40	21		5	5	5	5	5	6		5	5	5
*Dendropoma rhyssoconcha*	29	24	25						6			5			
*Serpulorbis variabilis*	13	25	15	8											
*Calcinus haigae*	51	21													
*Calcinus hazletti*	21	31		12		7		34		33			22		11
*Calcinus seurati*	9	30	78	44											
*Panulirus marginatus*		19	48				56		56	53	53	36	47	29	46
*Panulirus penicillatus*	46	43		47			5	33		5		18	30		
*Holothuria atra*	30		24	30	5			28			12		37	35	23
*Holothuria whitmaei*	26			33				26			22		57	24	59
*Acanthaster planci*	106	81	25	24	30			13					27		
*Montipora capitata	47	47	51			25		40		47	48	50	44	43	51
*Porites lobata	23		16			18	21	23	34	33			44	21	22
*Squalus mitsukurii*	7		91		8										
*Monachus schauinslandi*	________________54________________					7		766			656	310	260	134	222
*Stenella longirostris	79	59	40	32	45			33					47	119	51

Species with * show a significant isolation-by-distance signature across the archipelago.
